# The Effectiveness of Psychoeducational Interventions in Adolescents’ Anxiety: A Systematic Review Protocol

**DOI:** 10.3390/nursrep12010022

**Published:** 2022-03-15

**Authors:** Tânia Morgado, Vera Lopes, Dulce Carvalho, Eduardo Santos

**Affiliations:** 1 Pediatric Hospital of the Centro Hospitalar e Universitário de Coimbra, 3000-602 Coimbra, Portugal; lopes.vera@chuc.min-saude.pt; 2 Health Sciences Research Unit—Nursing (UICISA: E), Nursing School of Coimbra, 3000-232 Coimbra, Portugal; esantos@essv.ipv.pt; 3 Center for Health Technology and Services Research (CINTESIS), NursID, 4200-450 Porto, Portugal; 4 Hospital Sobral Cid, Centro Hospitalar e Universitário de Coimbra, 3040-714 Coimbra, Portugal; dulce.carvalho@chuc.min-saude.pt; 5 Nursing Research Group, Centro Hospitalar e Universitário de Coimbra (UICISA: E), 3004-561 Coimbra, Portugal; 6 Viseu Higher School of Health, Polytechnic Institute of Viseu, 3500-843 Viseu, Portugal

**Keywords:** adolescent, mental health, anxiety, early intervention, educational, systematic review

## Abstract

The COVID-19 pandemic had a strong impact on increasing anxiety in adolescents. This systematic review aims to identify the most effective psychoeducational interventions for reducing anxiety in adolescents following the guidelines of the Joanna Briggs Institute (JBI). The inclusion and exclusion criteria have been defined, and the search strategy has been planned. The search strategy will aim to locate both published and unpublished studies using, among other databases: CINAHL Plus with Full Text; PubMed; the Cochrane Central Register of Controlled Trials; and the JBI Database of Systematic Reviews. Following the search, all identified citations will be collated and uploaded into Endnote, and duplicates removed. Titles and abstracts will then be screened by two independent reviewers and by a third reviewer if a disagreement occurs. The results of the search will be reported in full in the final systematic review and presented in the PRISMA flow diagram. Eligible studies will be critically appraised for methodological quality using standardized critical appraisal instruments from the JBI. Data will be extracted from the studies included using the standardized JBI data extraction tool. For data synthesis, studies will be pooled using JBI SUMARI. The GRADE approach for grading the certainty of evidence will be followed, and a summary of findings will be created using GRADEPro GDT software. The results from this systematic review are expected to provide an overview of the most effective psychoeducational interventions for reducing anxiety in adolescents, allowing researchers to design and propose a new multicomponent psychoeducational intervention that will be validated and tested in the future. PROSPERO protocol registration number: CRD42020204356.

## 1. Introduction

At present, the 1.2 billion adolescents aged 10–19 years represent over 18% of the global population [1]. Adolescence, as a developmental transition [2] between childhood and adulthood, is a critical life stage for mental health, and several mental disorders peak in this phase [3,4].

Approximately 16% of children and adolescents suffer from some mental disorder [4,5,6], and several adolescent-specific factors make this age group particularly vulnerable to the development of anxiety [7]. The worldwide prevalence of any anxiety disorder in children and adolescents before the COVID-19 pandemic was estimated to be 6.5% [8,9]. Recently, several articles reported the effect of the pandemic on increased anxiety in adolescents [10,11,12,13,14,15,16,17,18], reporting about 38% of children and adolescents experiencing anxiety [13,17].

Anxiety refers to the brain’s response to danger or stimuli that an organism will actively attempt to avoid. This brain response is a basic emotion already present in infancy and childhood, and it is adaptive in many scenarios when it facilitates the avoidance of danger [19]. Anxiety becomes maladaptive when it interferes with functioning and becomes overly frequent, severe, and persistent. Thus, pathological anxiety can be characterized by persisting or extensive degrees of anxiety and avoidance associated with subjective distress or impairment. The differentiation between normal and pathological anxiety, however, can be particularly difficult in children because children manifest many fears and anxieties as part of typical development [19]. Anxiety disorders among adolescents are associated with impairment in academic, social, and family functioning and tend to have a chronic and unremitting course, persisting into adulthood [20].

Adolescence is also characterized by the development of a great capacity to think abstractly, to process information, and make maximum use of it. The capacity for reasoning corresponds to the stage of formal or operative-formal operations in Piaget’s theory of cognitive development, in which the adolescent constructs abstract and conceptual thinking, taking into account possible hypotheses, different points of view, and being able to think scientifically [21]. These characteristics make adolescence a great stage to promote mental health literacy [3,22,23] and for the development and implementation of psychoeducational interventions for anxiety.

Psychoeducation is the provision of systematic, relevant, broad, and up-to-date information about an illness or condition, including its diagnosis and treatment. Psychoeducational programs provide both disease-specific information (e.g., early recognition and management of relapse symptoms or any potential genetic implications of the illness) and general information (e.g., promotion of a healthy lifestyle, problem-solving, communication skills training, the identification of stressors in households, and education of family members and primary caretakers in their amelioration) [24]. Furthermore, psychoeducation includes information on how to explain aspects of living with an illness to family members so that they can understand the effect of the illness and assist the patient and treatment providers in the treatment program [24]. Psychoeducational interventions combine the elements of cognitive behavior therapy, group therapy, and education and its basic aim is to provide the patient and families knowledge about various facets of the illness and its treatment so that they can work together with mental health professionals for a better overall outcome [25]. More recently, some authors have reinforced the importance of developing psychoeducational interventions in the health–illness continuum and throughout the life cycle in different contexts [26,27], namely in children and adolescents, their families, and caregivers [28,29,30,31]. Some authors have assessed the effectiveness of psychoeducational interventions in increasing anxiety mental health literacy [26,32,33] and reducing anxiety levels in adolescents [34,35].

A preliminary search of the JBI Database of Systematic Reviews and Implementation Reports, the Cochrane Database of Systematic Reviews, PROSPERO, PubMed, MEDLINE, and CINAHL was conducted, and no current or underway systematic reviews on this topic were identified. This systematic review aims to identify the most effective psychoeducational interventions for reducing anxiety in adolescents. The questions of this review are: (1) What are the most effective psychoeducational interventions for adolescents in reducing anxiety? (2) What are the most effective psychoeducational interventions for adolescents in increasing self-control of anxiety? (3) What are the most effective psychoeducational interventions for adolescents in increasing anxiety mental health literacy?

## 2. Methods

**Figure 1:**
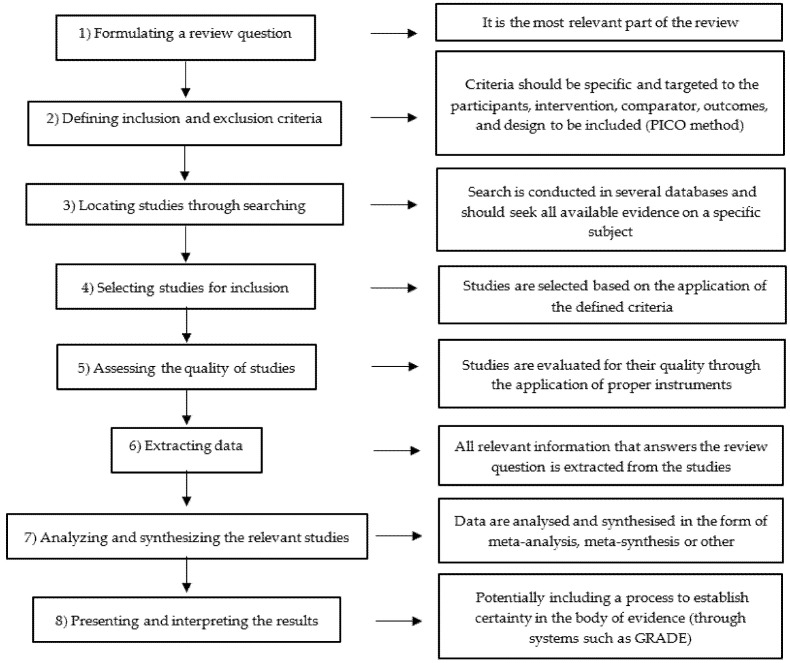
The JBI method and its procedures.

This systematic review will be conducted in accordance with the Joanna Briggs Institute (JBI) methodology for systematic reviews of effectiveness evidence [36]. The JBI is one of the international interdisciplinary collaborations (such as the Cochrane, the Campbell Collaboration, and others) that, given the growing interest in and production of systematic reviews, have promoted the standardisation of methods and, from there, promote the synthesis and implementation of science [37]. Currently, JBI has formal guidance for several types of reviews, such as systematic reviews of experiences, systematic reviews of effectiveness, systematic reviews of text and opinion, systematic reviews of prevalence and incidence, systematic reviews of costs of a certain intervention, process, or procedure, systematic reviews of etiology and risk, systematic reviews of mixed methods, systematic reviews of diagnostic test accuracy, umbrella reviews, and scoping reviews [37]. Generally, the JBI method proposes that the following steps are required in a systematic review of any evidence type: (1) Formulating a review question; (2) Defining inclusion and exclusion criteria; (3) Locating studies through searching; (4) Selecting studies for inclusion; (5) Assessing the quality of studies; (6) Extracting data; (7) Analyzing and synthesizing the relevant studies; and (8), Presenting and interpreting the results [37] (Figure 1).

This review was registered on PROSPERO with the protocol registration number: CRD42020204356. Accordingly, the inclusion and exclusion criteria and the design of the search strategy will be supported by the Population (P), Intervention (I), Comparator (C), and Outcomes (O) mnemonic.

### 2.1. Inclusion Criteria

#### 2.1.1. Participants

At the same time that adolescence represents a critical phase of life for mental health, and several mental disorders peak in this phase [3,4], it is simultaneously a privileged phase for the development of mental health literacy interventions [3,22,23] and psychoeducational interventions on anxiety. This review will consider all studies that include adolescents from 10 to 19 years who had participated in a psychoeducational intervention on anxiety. In this review will be exclusion criteria: another age, other diseases (e.g., chronic disease), and other health situations.

#### 2.1.2. Interventions

Psychoeducational interventions have been developed from promotion and prevention to treatment and recovery in mental health, namely in children and adolescents, their families, and caregivers [29,38,39,40]. This review will consider studies that evaluate the effectiveness of psychoeducational intervention on anxiety. For this purpose, according to the literature, it will consider education, psychoeducation, and cognitive behavior therapy. We will also consider the intervention feature in relation to “Who? What? When? Where? How?” There is no context limitation.

#### 2.1.3. Comparators

In the literature, we found other interventions that have contributed to the prevention, reduction, and management of anxiety in adolescents [41,42,43,44,45,46]. This review will consider studies that compare the psychoeducational interventions to other alternative non-pharmacological interventions (e.g., play therapy, mindfulness, psychodynamic, and others) and usual interventions or usual care.

#### 2.1.4. Outcomes

Psychoeducational interventions have been developed along the health–disease continuum and from prevention to treatment, seeking to prevent or reduce anxiety in adolescents and/or increase literacy [26,47,48]. This review will consider studies that include the following outcomes, measured by validated instruments: the primary outcomes of (1) reducing anxiety and anxiety disorders and (2) increasing self-control of anxiety; and the secondary outcomes of increasing anxiety mental health literacy.

#### 2.1.5. Types of Studies

This review will consider both experimental and quasi-experimental study designs, including randomized controlled trials, non-randomized controlled trials, before and after studies, and interrupted time–series studies. In addition, analytical observational studies, including prospective and retrospective cohort studies, will be considered for inclusion. This review will also consider descriptive observational study designs, including case series, individual case reports, and descriptive cross-sectional studies for inclusion. Studies published in the English, French, Spanish and Portuguese languages will be included, as well as studies published from 2010, because of the definition of adolescence (10–19 years) for the World Health Organization and the enlargement of pediatric age in Portugal. Before this date, studies in this area were few, and interventions were poorly described and with many methodological flaws.

### 2.2. Search Strategy

The search strategy will aim to locate both published and unpublished studies. An initial limited search of MEDLINE (PubMed) was undertaken (Appendix A) to identify articles on the topic. The text words are contained in the titles and abstracts of the relevant articles. This informed the development of a search strategy, including identified keywords and index terms, which were tailored for each information source. The search strategy, including all identified keywords and index terms, will be adapted for each included information source. The reference list of all studies selected for critical appraisal will be screened for additional studies.

### 2.3. Information Sources

We will use different information sources (e.g., electronic databases, contact with study authors, etc.). The databases to be searched include CINAHL Plus with Full Text; MEDLINE (PubMed); the Cochrane Central Register of Controlled Trials; the JBI Database of Systematic Reviews; Scopus; Psychology and Behavioral Sciences Collection; PsycINFO; and the Library, Information Science & Technology Abstracts (SciELO). Sources of unpublished studies and gray literature to be searched include: RCAAP—Repositório Científico de Acesso Aberto de Portugal; and OpenGrey—System for Information on Grey Literature in Europe e Banco de teses da CAPES (Brasil).

### 2.4. Study Selection

**Figure 2:**
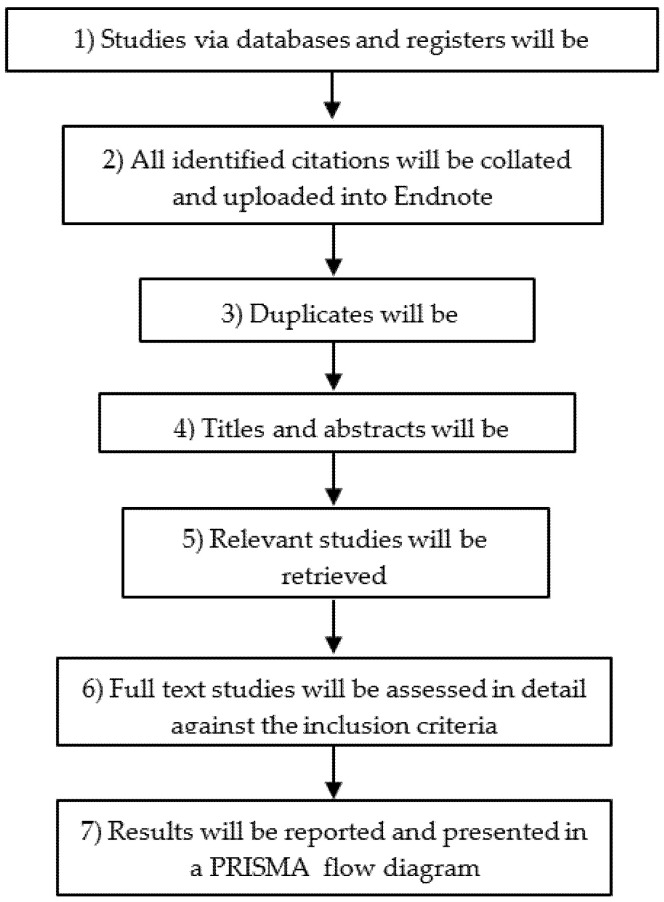
Study selection and its procedures.

Following the search, all identified citations will be collated and uploaded into Endnote, and duplicates removed. Titles and abstracts will then be screened by two independent reviewers for assessment against the inclusion criteria for the review. Potentially relevant studies will be retrieved in full, and their citation details imported into the Joanna Briggs Institute System for the Unified Management, Assessment and Review of Information (JBI SUMARI) [46,49]. The full text of selected citations will be assessed in detail against the inclusion criteria by two independent reviewers. The reasons for the exclusion of full-text studies that do not meet the inclusion criteria will be recorded and reported in the systematic review. Any disagreements that arise between the reviewers at each stage of the study’s selection process will be resolved through discussion or with a third reviewer. The results of the search will be reported in full in the final systematic review and presented in a Preferred Reporting Items for Systematic Reviews and Meta-Analyses (PRISMA) flow diagram [50,51] (Figure 2).

### 2.5. Assessment of Methodological Quality

Eligible studies will be critically appraised by two independent reviewers for methodological quality in the review using standardized critical appraisal instruments from the Joanna Briggs Institute for experimental and quasi-experimental studies. Authors of papers will be contacted to request missing or additional data for clarification, where required. Any disagreements that arise between the reviewers will be resolved through discussion or with a third reviewer. The results of the critical appraisal will be reported in narrative form and in a table. All studies, regardless of the results of their methodological quality, will undergo data extraction and synthesis.

### 2.6. Data Extraction

Data will be extracted from studies included in the review by two independent reviewers using the standardized Joanna Briggs Institute data extraction tool in JBI SUMARI [49]. The data extracted will include specific details about the populations, study methods, interventions, and outcomes of significance to the review objective. Any disagreements that arise between the reviewers will be resolved through discussion or with a third reviewer. Authors of papers will be contacted to request missing or additional data, where required.

### 2.7. Data Synthesis

Studies will, where possible, be pooled with statistical meta-analysis using JBI SUMARI [49]. Effect sizes will be expressed as either odds ratios (for dichotomous data) or weighted (or standardized), and final post-intervention mean differences (for continuous data), and their 95% confidence intervals will be calculated for analysis. Heterogeneity will be assessed statistically using the standard chi-square and the *I*^2^ tests. If the *I*^2^ index is ≤50%, the fixed-effect model will be selected to calculate the pooled effects; otherwise, a random effect model will be used [36]. Data will also be explored using subgroup analyses based on the different proposed interventions. Sensitivity analyses will be performed to test the robustness of the results and whether the quality of publication could influence the results. A funnel plot will be generated with STATA^®^ 14.0 software (StataCorp LP: College Station, TX, USA) to assess publication bias if there are 10 or more studies included in a meta-analysis. Statistical tests for funnel plot asymmetry (Egger test, Begg test, and Harbord test) will be performed, where appropriate. Where statistical pooling is not possible, the findings will be presented in narrative form, including tables and figures to aid in data presentation, where appropriate.

### 2.8. Assessing Certainty in the Findings

The Grading of Recommendations, Assessment, Development and Evaluation (GRADE) approach for grading the certainty of evidence will be followed, and a Summary of Findings (SoF) will be created using GRADEPro GDT software (Evidence Prime Inc., Hamilton, ON, Canada). The SoF will present the following information where appropriate: absolute risks for the treatment and control, estimates of relative risk, and a ranking of the quality of the evidence, based on the risk of bias, directness, heterogeneity, precision, and risk of publication bias of the review results.

## 3. Expected Results

With this review, we aim to identify the most effective psychoeducational interventions for reducing anxiety in adolescents, increasing anxiety mental health literacy, and anxiety management and control. Another implication of this study for practice and research is that it will allow researchers to design and propose a new multicomponent psychoeducational intervention that will be validated and tested in the future. With that psychoeducational intervention, we hope to enable adolescents to recognize, prevent and manage anxiety and ask for help which will facilitate early identification and mental health intervention.

## Appendix A

**Table A1:** Draft of search strategy to Medline (PubMed) ^1^.

Search No.	Query	Records Retrieved
#1	Search (((((((((((((((((((((((psychoeducation *[Title/Abstract]) OR psychology[MeSH Terms]) OR psycholog *[Title/Abstract]) OR clinical psychology[MeSH Terms]) OR process, psychotherapeutic[MeSH Terms]) OR psychotherap *[Title/Abstract]) OR psychotherapy[MeSH Terms]) OR group psychotherapy[MeSH Terms]) OR group psychotherapy *[Title/Abstract]) OR cognitive behavior therapy[MeSH Terms]) OR cognitive behavior therap *[Title/Abstract]) OR behavior therapy[MeSH Terms]) OR behavior therap *[Title/Abstract]) OR cognitive psychotherapy[MeSH Terms]) OR cognitive psychotherap *[Title/Abstract]) OR therapeutics[MeSH Terms]) OR therapy *[Title/Abstract]) OR play therapy[MeSH Terms]) OR play therap *[Title/Abstract]) OR mindfulness[MeSH Terms]) OR mindful *[Title/Abstract]) OR education[MeSH Terms]) OR education *[Title/Abstract]) OR psychodynamic *[Title/Abstract]	6,651,967
#2	Search ((((((((((((anxiety[MeSH Terms]) OR anxiet *[Title/Abstract]) OR anxiety disorder[MeSH Terms]) OR phobia, social[MeSH Terms]) OR social phobi *[Title/Abstract]) OR phobic disorder[MeSH Terms]) OR phobi *[Title/Abstract]) OR panic[MeSH Terms]) OR panic *[Title/Abstract]) OR anxiety disorder, separation[MeSH Terms]) OR separation anxiet *[Title/Abstract]) OR agoraphobia[MeSH Terms]) OR agoraphobi *[Title/Abstract]	254,251
#3	Search (adolescent[MeSH Terms]) OR adolescen *[Title/Abstract]	2,021,158
#4	Search ((((adolescent[MeSH Terms]) OR adolescen *[Title/Abstract])) AND (Search (((((((((((((((((((((((psychoeducation *[Title/Abstract]) OR psychology[MeSH Terms]) OR psycholog *[Title/Abstract]) OR clinical psychology[MeSH Terms]) OR process, psychotherapeutic[MeSH Terms]) OR psychotherap *[Title/Abstract]) OR psychotherapy[MeSH Terms]) OR group psychotherapy[MeSH Terms]) OR group psychotherapy *[Title/Abstract]) OR cognitive behavior therapy[MeSH Terms]) OR cognitive behavior therap*[Title/Abstract]) OR behavior therapy[MeSH Terms]) OR behavior therap *[Title/Abstract]) OR cognitive psychotherapy[MeSH Terms]) OR cognitive psychotherap *[Title/Abstract]) OR therapeutics[MeSH Terms]) OR therapy *[Title/Abstract]) OR play therapy[MeSH Terms]) OR play therap *[Title/Abstract]) OR mindfulness[MeSH Terms]) OR mindful *[Title/Abstract]) OR education[MeSH Terms]) OR education *[Title/Abstract]) OR psychodynamic *[Title/Abstract])) AND (Search ((((((((((((anxiety[MeSH Terms]) OR anxiet*[Title/Abstract]) OR anxiety disorder[MeSH Terms]) OR phobia, social[MeSH Terms]) OR social phobi*[Title/Abstract]) OR phobic disorder[MeSH Terms]) OR phobi *[Title/Abstract]) OR panic[MeSH Terms]) OR panic*[Title/Abstract]) OR anxiety disorder, separation[MeSH Terms]) OR separation anxiet *[Title/Abstract]) OR agoraphobia[MeSH Terms]) OR agoraphobi *[Title/Abstract])	365

^1^ Search date: 13 December 2021.
